# Effect of temperature-control liner materials on long-term outcomes of lower limb prosthesis use: a randomized controlled trial protocol

**DOI:** 10.1186/s13063-019-3920-4

**Published:** 2020-01-10

**Authors:** Goeran Fiedler, Anita Singh, Xueyi Zhang

**Affiliations:** 1 Department of Rehabilitation Science and Technology, School of Health and Rehabilitation Sciences, University of Pittsburgh, Pittsburgh, PA USA; 2 Department of Biomedical Engineering, School of Engineering, Widener University, Chester, PA USA

**Keywords:** Artificial limbs, Prosthesis materials, Prosthesis utilization

## Abstract

**Background:**

In people living with limb loss, addressing the resulting functional deficit with prostheses increases the risk for secondary conditions such as pressure sores, impaired blood perfusion, and injuries from accidental falls. Any of those occurrences can render the prosthesis temporarily useless, making it challenging for users to engage in many activities of daily life, including work, exercise, and social participation. Many of the described issues originate at the interface between residual limb and prosthetic socket, where the objectives of sufficient weight distribution and suspension are conflicting with the necessity to facilitate heat exchange and limit contact pressure and friction.

Recently, prosthesis liners that contain phase-change material have become commercially available, holding the promise that the micro climate at the interface between the residual limb skin and the prosthetic socket can be regulated to reduce the users’ tendency to sweat. Preliminary studies on these liners indicate that the socket temperatures inside the socket stayed lower and rose slower than in conventional liners. However, the clinical relevance of those findings remains unclear.

The purpose of this study is to investigate whether longer (6+ months) periods of use of phase-change material based temperature-control liners have clinically meaningful effects.

**Methods:**

The protocol is a double-blind longitudinal cross-over research design. A sample of trans-tibial prosthesis users are wearing their regular gel or silicone liners for six months and phase-change material liners for another six months in a randomized sequence. Their prostheses is equipped with activity monitors to detect days when they could not wear their prosthesis. In six-week intervals, individuals’ activity, physical performance, and overall prosthesis assessment is recorded using standardized methods.

**Discussion:**

Expected results will inform prescription and reimbursement practice of phase-change material-based prosthesis liners and will help improve and economize prosthetic fitting for people with limb loss. The design and duration of the protocol, including randomization, blinding, and within-subject comparison, will generate scientific evidence of a comparably high level. Inclusion of a comparably large sample and different climates, e.g. across all four seasons, will make findings applicable to a large number of prosthesis users.

**Trial registration:**

Clinicaltrials.gov, NCT03428815. Registered on 12 February 2018.

## Background

The (partial) loss of a lower limb is followed by many undesirable consequences. Beyond the immediate and obvious impairments of physical integrity and function, which are addressed with prostheses, a number of potentially severe secondary health effects are of concern. Those include pressure sores [1], joint contractures [2], muscle atrophy [3], impaired blood perfusion [4], and injuries from accidental falls [5]. The necessary containment in a tight-fitting prosthesis socket and the resulting excessive contact and shear forces may lead to tissue breakdown within the residual limb. This, in turn, can render the prosthesis temporarily useless as it cannot be worn while the residual is sore. In such instances, prosthesis users are challenged to engage in many activities of daily life, including work, exercise, and social participation, which leads to well documented detrimental effects on physical and psychological health [6].

Prosthesis suspension by liner is a very prevalent technique especially in trans-tibial prosthetics, where it is considered the standard of care; a sized flexible liner from silicon or polyurethane gel is rolled up on the residual limb, providing excellent adhesion to the skin. The user then enters the rigid prosthesis socket where the liner is anchored either with a pin lock system or by means of a vacuum. While this system combines good prosthesis suspension with comfortable donning and doffing, many users complain about excessive sweat accumulation and subsequent problems with slippage, skin irritation, and discomfort [7–9].

The problem of insufficient heat exchange in the prosthetic socket has been addressed by a number of research and development projects [10–12]. Published works include studies on sockets that were structurally modified with cooling channels [13] or thermoelectric elements based on the Peltier effect [14], achieving a desired cooling effect either way. However, none of the described technologies is advanced enough to facilitate clinical trials and outcome assessments beyond mere temperature measurement.

Phase-change materials (PCMs) have the capability to absorb thermal energy by changing from solid to liquid phase. They have been successfully used for countless temperature-control applications, including in space crafts, textiles, computer cooling, and others [15]. Recently, prosthesis liners that contain PCM have become commercially available, holding the promise that the microclimate at the interface between the residual limb skin and the prosthetic socket can be regulated to reduce the users’ tendency to sweat. Marketed under the name “Smarttemp” by the Ohio Willow Wood Company (Sterling, OH, USA), these liners have essentially the same indications and contraindications as conventional liners but are slightly more expensive than traditional silicon or gel liners. A recent double-blind randomized study with 16 trans-tibial prosthesis users indicated that socket temperature and perspiration inside the socket stayed lower and rose slower in PCM-based liners than in conventional gel liners if measured > 1 h after individuals used a stationary bike for 25 min [16].

However, the clinical relevance of those findings remains unclear. While (perceived) socket comfort is certainly an important criterion in prosthesis fitting, it may be claimed that only tangible functional benefits are of concern. It remains particularly questionable whether the reduced skin temperature will indeed lead to better socket suspension, more effective utilization of the prosthesis, and consequently to increased activity, better overall health, and greater quality of life for the user. As the known previous study only assessed the capabilities of temperature-control mechanisms at discrete timepoints, it is unknown whether a clinically significant cooling effect can be sustained over longer periods of time and whether there are unforeseen side effects of the interventions, such as a change in the mechanical properties of the material that could lead to socket misfit and to imperfect contact pressure distribution.

Previously published liner comparison studies investigated outcomes after wearing times of two weeks [17], three weeks [18], four weeks [19], and 2.5 months [20] and have been criticized for short accommodation times and the resulting lack of firm conclusions [21].

A comprehensive outcome variable of high importance is prosthesis utilization, measured in time per year. Involuntary non-use of the prosthesis will severely affect a person’s mobility and ability to participate in activities of daily life [22–24]. This is an issue both on the personal level [25] and on a larger economic level, as increased absenteeism from work or loss of productivity while at work [26] cause a large part of the indirect costs associated with disability [27, 28]. Weekly or daily wear times have been frequently reported as a measure of use of and satisfaction with lower limb prostheses [29]. Depending on factors such as time since amputation, rehabilitation program, and cause of the limb loss, those wear times are in the range of 40 [30] to 80 [7] h per week.

Physical performance and prosthesis-related quality of life are likewise closely related to socket comfort [31–33] and are expected to be affected by the liner material. An uncomfortable or poorly suspended socket may trigger any degree of altered gait biomechanics to compensate for the deficits in order to, for instance, relieve a pressure sensitive area [34] on the residual or to reduce accelerations [35] that lead to displacements between residual limb and prosthesis. Those compensations are by nature less energy-efficient than normal walking [36–38] and will lead to slower gait velocities and overall to lower levels of physical performance. The compensations may also not be entirely effective in avoiding the undesirable consequences of poor socket comfort, such as pain and gait instability. This has been reflected by self-reported quality-of-life ratings [6, 39, 40].

In summary, excessive sweating in a prosthesis socket is a recognized problem with several adverse consequences. PCM liners have been introduced to provide better temperature control, but their clinical effects have not yet been thoroughly studied.

The purpose of the following protocol is therefore to investigate whether the use of PCM-infused liners can increase activity level, gait efficiency, and prosthesis utilization over longer (6+ months) periods of use. The research question follows the rationale that lower and steadier skin temperatures should result in reduced sweat, friction, skin damage, and under-utilization of the prosthesis. This would encourage users to wear their prosthesis for longer periods of time and for an expanded array of purposes, thus increasing their ability to ambulate and to engage in a greater variety of activities. This in turn would improve their overall health—including, but not limited to, cardiovascular health, musculoskeletal health, and mental health—collectively signified by increased quality-of-life ratings.

### Specific aims/hypotheses

Specific aims include:

1. To compare PCM liners to the conventional liners with regard to activity and participation;
  *Hypothesis 1a (primary hypothesis):* Use of PCM-based temperature-control liners will improve prosthesis utilization (measured in self-reported days of prostheses use per year) when compared to conventional liners;
  *Hypothesis 1b (secondary hypothesis):* Use of PCM-based temperature-control liners will improve prosthesis utilization (measured in step activity over time) when compared to conventional liners;
2. To quantify the short- and long-term effects of PCM liners on activity, health-related quality of life, and performance;
  *Hypothesis 2a:* Use of PCM-based temperature-control liners will improve activity (signified by number and distribution of daily steps), physical performance (measured by 2-min walk test [2MWT]), and self-reported prosthesis-related quality of life (assessed by questionnaire) when compared to conventional liners over the course of six weeks;
  *Hypothesis 2b:* Use of PCM-based temperature-control liners will sustainably improve activity (signified by number and distribution of daily steps), physical performance (measured by 2MWT), and self-reported prosthesis-related quality of life (assessed by questionnaire) when compared to conventional liners over the course of six months;
3. To investigate the relationship between perceived benefits of PCM liners and patient-centric outcomes;
  *Hypothesis 3:* Differences in subjective ratings of prosthesis satisfaction (assessed by questionnaire) between PCM-based temperature-control liners and conventional liners will positively correlate with respective differences in objective data on prosthesis utilization (average daily step counts) and physical performance (2MWT).

## Methods

The protocol has been approved by the responsible Institutional Review Boards. Study participants are asked by the principle investigators to provide informed consent in accordance with human-subjects protection regulations. The protocol has been designated as low risk and no separate oversight committee has been instituted. No sensitive or identifiable data will be collected, which reduces the risks associated with breaches of confidentiality. Therefore, no data monitoring committee has been mandated by the bodies reviewing and approving the protocol.

### Participants

Participants are recruited from the community at two sites, in Chester, PA, and Pittsburgh, PA. Recruitment flyers are posted in the waiting areas of physicians’ offices, prosthetics businesses, and a prosthetics educational institution. The study is further advertised in online (university research registry) and print materials (newspaper), and at local events frequented by prosthesis users (e.g. amputee support group meetings). Potential participants are asked to contact the investigators by phone or set up a screening call in person or by online form. During the call, eligibility is determined and, if appropriate, an intake appointment is scheduled. Inclusion criteria are the use of a lower-limb prosthesis with liner suspension, at least one year of prosthesis use, a well-fitting socket, the ability to walk with the prosthesis outdoors without notable limitations (K-Level 3), stable weight, and absence of acute medical conditions that would temporarily affect the ability to use prostheses. Exclusion criteria are use of a non-standard liner size, known allergies against liner materials, and any inability to understand the protocol and to comply with the associated tasks, such as maintaining a log of days when the prosthesis could not be used.

Power analysis was based on the cross-over statistical methods above and previous literature and one-sided determination. Halsne et al. [41], in an analysis of 12-month step activity data in participants with trans-femoral amputation, reported that the average change in step activity scores for individuals in the hot and cold months of the year was significantly higher than in the moderate temperature months of the year corresponding to an effect size of 0.47. Since PCM liner material mitigates the residual limb temperature fluctuations and sweating that may be at the root of those differences, a similar standardized effect size was assumed for the study intervention. The intervention periods of six months each cover both hot and cold seasons, leading to the assumption that underlying conditions are comparable to the Halsne study. This effect size, and a sample size of 50 participants, results in 85% power to detect significant differences.

To illustrate the clinical significance of such an effect, the assumed effect size would conservatively equate a difference in prosthesis utilization of four days per year if the average yearly number of days with prosthesis use has a standard deviation of 8.5 days. Irrespective of the statistical significance of such an effect, every day of work absence has a relevant economical effect to the employee and the employer [42]. Given the duration of the study and the potential reluctance of subjects to change their liner type, it is anticipated that up to 9 (18%) participants do not complete the protocol, bringing the statistical power to 80%. Comparable prospective intervention studies in the past have utilized sample sizes between nine [43], 13, [20] and 20 [44].

A steady state within either of the interventions will be assumed [45], yet it will be possible to detect any baseline drift or seasonal fluctuations with the proposed repeated measures design. Previous studies have used a repeated measures design to investigate the reliability of outcome measures, such as the Locomotor Capabilities Index and the 2MWT [46–48]. The Prosthesis Evaluation Questionnaire (PEQ) has been validated in that fashion as well [49].

Missing values are possible due to drop-out and intermittent non-compliance with the data collection schedule. This would not invalidate “year-on-year” comparison of the remaining datasets. Provided that baseline characteristics can be sufficiently described by existing data, it may also be possible to interpolate and extrapolate missing data points with some accuracy.

Despite the long duration of the data collection, the intervention itself will have a comparably low burden on the individuals’ daily lives, which is expected to make participation appealing to a wide range of potential participants who are targeted by online and offline postings.

### Methods

**Fig. 1:**
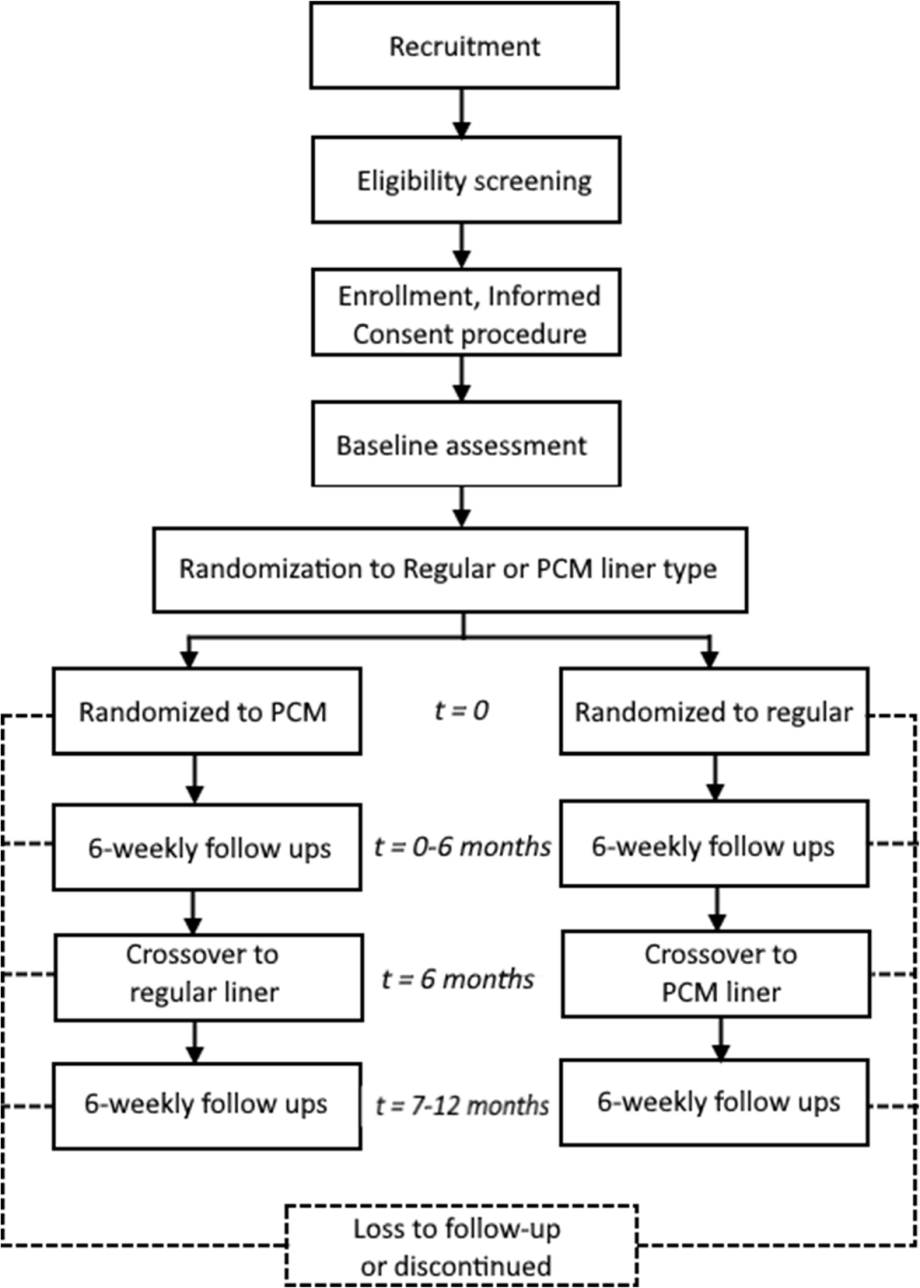
Flowchart illustrating the protocol timeline

The protocol utilizes a double-blind longitudinal cross-over superiority design. A sample of trans-tibial prosthesis users is randomly assigned to one of two groups with an allocation ratio of 1:1. While individuals in one group receive PCM liners, the members of the other group are fitted with regular gel or silicone liners. After six months, the first group receives conventional liners (and turn their PCM liners in) and the second group receives PCM liners (Table 1 and Fig. 1). Prostheses in either group are equipped with activity monitors and participants are asked to keep daily notes of any perceived issues with their residual limb or socket fit. In regular intervals, individuals’ physical performance capabilities and prosthesis-related quality of life, including limb and overall health, are assessed using standardized methods.

**Table 1:**
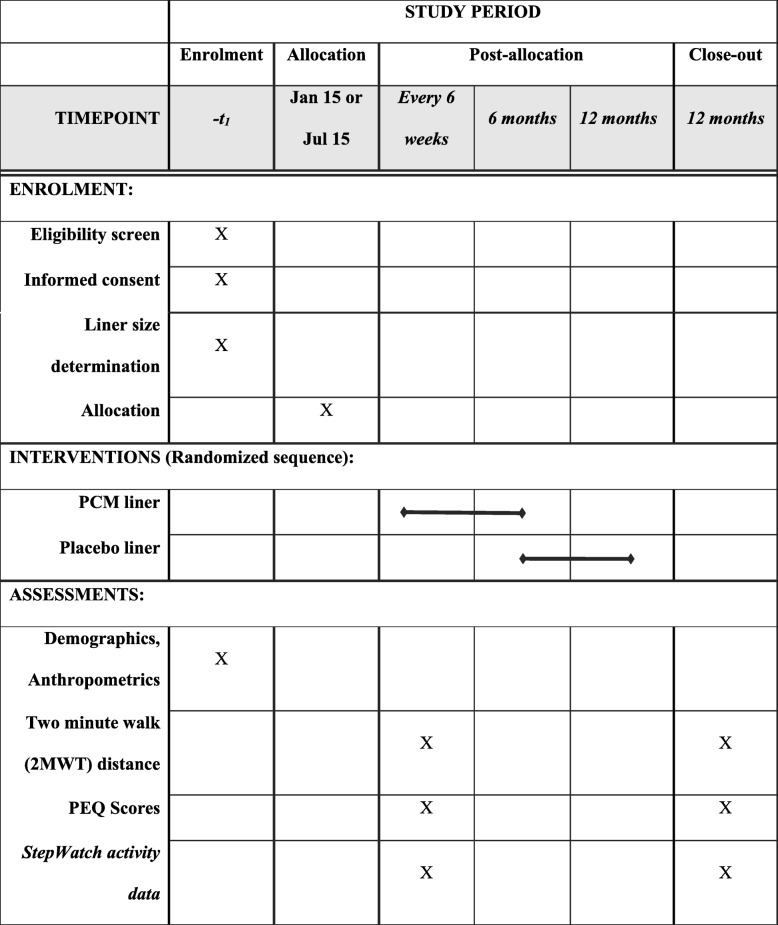
Schedule of enrolment, interventions, and assessments. *2MWT* Two Minute Walk Test, *PEQ* Prosthesis Evaluation Questionnaire

Comparing liners over the duration of six months allows detecting short- and long-term effects of the different approaches, including effects due to seasonal outdoor temperature changes, material abrasion and fatigue, and potential unsustainable material properties (i.e. diminished temperature-control capabilities of the PCM). In order to cover a comparable portion of the year regarding climatic conditions, the protocol is started at one of two dates only: mid-Winter (around 15 January) or mid-Summer (around 15 July). Any possible washout period after changing liners is expected to be completed well within the first assessment intervals [20], so that unbiased data can be compared for all the listed assessment points for either liner.

Randomization is performed by the study statistician, using proc. survey select available in SAS version 9.4. in a block randomization scheme with lengths of 2 and 4. Blocks length is unknown to the clinical personnel. This block design assures a balanced allocation in the groups and reduces the chance that testing personnel will be able to guess the next intervention group assignment. This, in turn, serves to minimize bias in participant allocation to the intervention group at the beginning of the study. No stratification factors are used. To facilitate group allocation, a table with liner serial numbers that only indicates A (for first intervention) and B (for second intervention) along with the individual identifier number will be shared via email by the statistician. Since all the study liners are made to look identical (apart from the serial numbers), investigators working off the table provided by the statistician have no way of telling the liner type. They will retrieve any assigned liner from storage and administer it to the participant solely by serial number.

Upon entering the study, every participant receives two identical new liners that match their currently used one in size as measured by a credentialed prosthetist. The liner material is either the same as in their existing liner or PCM-infused silicon, depending on group allocation. In order to blind the individuals, their care providers, data analysist, and study personnel to the treatment, all study liners are custom ordered for each participant from the same manufacturer (Willowwood, Sterling, OH, USA) in a uniform color and outer fabric design. The packaging is uniform as well, apart from a label containing the study participant number provided at ordering. Every liner is imprinted with a unique serial number that is the only sign by which it can be identified as a PCM or conventional liner. The manufacturer provides a list of these numbers, which will only be accessed by research personnel once the data collection and analysis procedures are completed, so that the results can be attributed to the proper group. There is no expectation that unblinding during the study could become necessary, as the intervention is not considered vital to participants. Upon conclusion of the study, participants will be told their individual sequence of interventions, which may inform their future choice of prosthesis liner. Two identical liners are provided to allow individuals to alternate between them on a daily basis, which is recommended practice in order to allow cleaning and air-drying liners and to maximize the lifetime of the material. (A regular life time of six months continuous wear is common for prosthesis liners [50].) Participants are instructed to handle and rotate the liners in their usual way, in order to assure realistic test conditions, but are asked to not wear their previously existing liner for the duration of the study.

The prosthesis-related quality of life is assessed during this first appointment, using domains “About the prosthesis” (question group 1 of the PEQ [51]), “Specific Bodily Sensations” (group 2), “Ability to Move Around” (group 4), and “Satisfaction with particular Situations” (group 5). “Importance of different aspects” (group 7) is assessed as well, to provide insights for appropriate interpretation of responses. The PEQ contains a large number of questions that have been devised to cover all aspects of prosthesis-related quality of life. The explicit intention of the tool is to provide the option of customizing a questionnaire by selecting a subset of the PEQ questions, all of which are considered equivalent and are scored on an analog scale of 0–100 [52]. The PEQ is valid for lower-limb prosthesis users and has high internal consistency (Cronbach’s alpha = 0.73–0.89) and temporal stability over a mean retest period of 30 days (intraclass correlation coefficient = 0.79–90) [51]. The likewise administered 2MWT [47] has well-established psychometric properties in lower-limb prosthesis users [48]. Of particular interest to the proposed study is the responsiveness of the 2MWT to rehabilitation in lower-limb prosthesis users [47]. Significant increases in total distance were found from initial prosthesis fitting (baseline) to discharge from rehabilitation and at a three-month follow-up [47]. A Stepwatch monitor (Modus Health, Washington, DC, USA) is affixed to the ankle of the prosthesis and measures step counts over time. This device has been utilized for a large number of research studies involving lower limb prosthetics [20, 53, 54] and meets the specifications issued by the Department of Veterans Affairs regarding outcome assessment technology [55]. Using the “Trex” evaluation algorithm, the device allows an assessment of user activity by considering ambulation energy, peak performance, and cadence variability indices.

In addition to determining liner sizes, participants’ height, weight, age, and time since limb loss are recorded at the first appointment.

Participants are asked to continue their regular activities of daily life while wearing the new liner and to make a note of any days during which the prosthesis could not be used due to residual limb health issues, as a redundancy to the step counter data. About every two weeks, a member of the research team will schedule a 5-min appointment with the participant to read out Stepwatch data. Individuals are not required to come to the research site for the respective appointments, which may be scheduled at their homes or places of work according to individuals’ preferences. While the Stepwatch monitor can store up to 50 days of data, two-week intervals have been chosen to mitigate the adverse effects of possible equipment malfunction or application errors. Malfunctioning step counters are expected to be the main risk for disagreement between self-report and step count data. Removing those data points from the total for analysis will effectively shorten the intervention period, but not to a degree substantial enough to challenge the underlying assumptions for the power analysis (e.g. malfunctioning step counters can be replaced at the next data collection point, limiting data loss to two weeks at most). Any adverse events or other unsolicited reports will be recorded as they occur. No scheduled auditing is planned. In the event that the protocol has to be amended, the required ethics approvals will be obtained and the updated version of the protocol will be shared electronically with the study personnel at both sites, the sponsor, clinical trial registry, and, if mandated by ethics review, with study participants.

Every six weeks, appointments are scheduled to conduct the 2MWT, complete sections “About the Prosthesis,” “Specific Bodily Sensations,” “Ability to Move Around,” “Satisfaction with Particular Situations,” and “Importance of Different Aspects” of the PEQ, and read out step data.

At the six-month appointment, after completing 2MWT and PEQ, individuals turn in their two study liners and receive two new liners, representing the respectively other variety. Following a short rest, participants will complete the 2MWT and PEQ once more before the test day is concluded while wearing the new liner.

The 12-month appointment marks the end of the data collection for this study. Individuals blinded to liner type will perform all data collection.

Participants receive a compensation of US$30 per site visit, for a total compensation of US$270. This payment schedule is intended to reduce the occurrence of drop-outs.

In the event of any unexpected discomfort or skin health issues, participants can discontinue or pause their participation at any point, simply by not wearing the study liner anymore and reverting to their original liner. The stated reason for discontinuation or pausation will be noted; data collected up to that point will be included in analyses, if possible (i.e. if paired data points for both interventions have been collected). Participants who merely pause their use of the study liners are subjected routinely to the to the regular follow-up schedule in order to obtain outcome data for intention-to-treat analyses. In participants who drop out entirely and are not available for follow-up, no additional data will be collected.

### Outcome variables

The main outcome variable is prosthesis utilization and is operationally defined as the “number of days with prosthesis use per time,” prorated to a full year. Prosthesis utilization is the clinically most relevant variable, as it describes the extent to which a person’s limb loss affects their social and economic participation [6]. To determine the number of days of prosthesis use, self-reported information will be evaluated along with step count data. A day without substantial prosthesis use will be assumed if the daily step count is below the 20th percentile for the average daily step count of the respective individual over the full year. Only if both methods agree on the number of dates without prosthesis will the information be used for analysis in order to reduce the errors caused by inaccurate memory or by malfunctioning equipment.

Secondary outcome variables will be “Average daily step count,” “Step count bouts per time unit,” “Two-minute walk distance,” “Prosthesis function rating,” “Wellbeing rating,” “Mobility rating,” and “Satisfaction rating,” the latter four of which are based on question groups 1, 2, 4, and 5 of the PEQ, respectively. Analyzing those variables will allow a more comprehensive understanding of the effects of PCM liners and may inform the hypotheses of subsequent studies investigating their function mechanisms.

Depending on the eventual sample composition, it may be possible to extract incidental findings on the effects of demographic and anthropometric factors on measured outcomes with the different liners. Any such results may be useful as pilot data for subsequent studies to further investigate the utility of PCM in prosthetics.

Several possible comparison variables are deliberately not included in this clinical trial. Monitoring changes in liner temperature or material properties during use would necessitate the use of additional data collection equipment, which would be likely to interfere with the regular use of the liners and thus pose a bias to the measured data. In addition, it is unclear how temperature data should be interpreted (e.g. whether lower temperatures are always better than higher or steady temperatures better than fluctuating) to become clinically meaningful. It is anticipated that the findings of the present study motivate subsequent research to investigate the function mechanisms of PCM liners and to better explain the results of this study.

Skin health, although an important outcome of prosthesis use, has likewise not been included in the study protocol. Assessment of residual limb skin health may help detect a number of adverse reactions to prosthesis use, ranging from skin abrasion to tissue necrosis. Measuring skin health outcomes results is fairly subjective and requires timely notification and assessment as skin issues occur. It is thus challenging to apply a reliable measure consistently across time and especially across different research sites. This study focuses on prosthesis utilization and considers skin and other health issue to have effects on the measured quality and quantity of prosthesis utilization. For the purposes of this research, the mechanisms that may lead to changes in that outcome variable are not the focus and investigating them is therefore deferred to subsequent studies.

### Statistical plan and data analysis

Statistical methods, as detailed below, will be applied to the main outcome variable “days of prosthesis use,” as well as the secondary variables. Exploratory data analysis will be conducted before formal statistical analysis. The distributions of all key variables will be examined to check for data errors and ensure that modeling assumptions are not violated. Numerical summaries including means, standard deviations, medians, and histogram graphical techniques will be used for continuous variables and frequencies will be computed for categorical variables. No interim analyses are planned.

#### Analysis of primary outcomes

Intervention effects on the primary outcome variable will be analyzed using the available cases (excluding missing data points) by comparing group means in a general linear model.

#### Analysis of secondary outcomes

Outcome data of the variables “Average daily step count,” “Step count bouts per time unit,” “Two-minute walk distance,” “Prosthesis function rating,” “Wellbeing rating,” “Mobility rating,” and “Satisfaction rating” are collected at baseline, 1.5 months, 3 months, 4.5 months, and 6 months and repeated at the second half-year. The effect of intervention, time, and group*time interactions will be evaluated. Baseline data for outcome variables will be compared between randomization groups and, if they are different, a random intercept mixed effect will be used. Mixed-effects modeling techniques will be used for the repeated measures. The Kolmogorov–Smirnov test will be used to verify the normality assumption and if it is violated appropriate transformations will be considered.

Correction for multiple testing will be by adjusting *p* values using the Benjamini–Hochberg method [56]. Before formal statistical analysis, data will be compared across treatments to check if they are comparable. If differences exist, baseline demographic effects will be taken into account in mixed effects models. The correlation among repeated measures will be examined and adjusted appropriately such as unstructured, confound symmetry, or auto-regressive.

#### Treatment of missing data

Data will be analyzed according to study group membership regardless of study completion. Since mixed-effects models do not assume a balanced design, participants who do not have data (drop-outs) on all 10 timepoints will still be included in the analysis (intent to treat). It will be tested if scores for the intervention are significantly different from the control condition’s scores for the primary outcome. A sensitivity analysis will be performed subsequently, re-running the analysis only using cases with complete data. In the event that only a small amount of data is missing, the missing values will be imputed. However, missing data patterns will be examined and reasons described for attrition and missing data because such patterns may be informative for identifying systematic biases in future studies. A missing data analysis of missing-at-random versus complete-at-random will be performed. Additionally, a missing-not-at-random analysis will be conducted to evaluate the respective implications for result interpretation. Covariates to include in this analysis are length of prosthesis experience, wellbeing rating at baseline, and satisfaction rating at baseline.

#### Determination of covariates

Patterns of association between descriptive measures (i.e. demographic and diagnosis characteristics) and intervention outcomes will be examined using paired t-tests, Chi-squared tests with Fisher’s exact, correlation analyses (and regression analyses, as appropriate). From these analyses, critical demographic and clinical characteristics will be identified that will be controlled for in the statistical models and incorporated into the design of future controlled clinical studies. In primary analysis, co-variables age, body weight, and preferred gait velocity (as determined by baseline 2MWT) will be controlled for. Other co-variates that will be evaluated and controlled for if necessary are length of prosthesis experience, amputation cause, and method of prosthesis suspension.

### Exploratory analyses

Possible additional analyses include a more detailed investigation of prosthesis utilization over time by comparing seasons, months, weekdays, or hours of day within and across interventions [41]. Furthermore, it may be possible to analyze covariates that predict prosthesis utilization, including anthropometric and demographic data.

### General considerations

Data management and analysis utilize a secure network and computer system to protect confidential data with security measures established by the HIPAA Security Rules. The data collected are verified, edited, and updated to provide clean data for analysis. Once data pass all the edit procedures, analytical datasets will be created for analysis by a statistician. As a final step in quality control and an initial step in analysis, descriptive statistics will be calculated and graphic displays created.

A table containing the SPIRIT checklist is provided as Additional file 1.

## Discussion

As a chronic condition, limb loss has the potential to trigger a person’s wellbeing in a number of different ways, including impaired function, pain and discomfort, higher rates of accidental falls (especially in lower limb loss) as well as untimely tissue degeneration of the existing bodily structures due to overuse, un-physiological postures, and (micro-) trauma. One co-morbidity that is almost universally experienced by any user of prosthetic limbs at some point is skin breakdown. The micro-climate at the skin–socket interface in combination with the long daily wear times explain the high likelihood of suffering skin abrasions, pressure sores, and infections. In comparison to people with vascular limb loss, who are often sedentary, young and active prosthesis users are likely to use their prostheses more—essentially every waking hour—and for more strenuous activities, thus increasing the perspiration and the friction between skin and socket.

In elderly and less active patients, the adverse effects of sweating in the prosthesis may not be as pronounced, but they are a concern nonetheless. In order to avoid many of the conditions that are associated with a sedentary lifestyle, it is important to facilitate a certain baseline of physical activity in people with a disability. Comfortable and well-fitting prostheses are an important element of such efforts.

If successful, we expect this research to provide an answer on how well PCM liners increase prosthesis use and activity in people with lower-limb loss. The level of evidence that can be achieved with the proposed randomized double-blind cross-over trial will allow for solid conclusions regarding the advantages (or absence thereof) of those newly introduced liners. If it is found that wearing PCM liners makes users substantially less likely to endure days without prosthesis use or to restrict their activities otherwise when, this would support their widespread clinical use in the future. If no such gains are evident, it could be concluded that any perceived improvement would be due to a placebo effect and therefore not supportive of changing established practices of liner prescription. In either case, our research will help avoid providing prosthesis users with less-than-optimal solutions, which will contribute to their treatment success and overall health outcomes.

Findings and data will be disseminated upon completion of the study, as conference abstracts, journal manuscripts, and final report to the study sponsor. Authorship eligibility will be determined following the guidelines of the International Committee of Medical Journal Editors.

This protocol, which has already been shown to be feasible in a pilot data collection, can easily be adapted to test similar interventions in future studies. A number of promising approaches addressing moisture control and wear comfort in prosthesis liners have recently been proposed, including active heat exchangers, perforated liners, and semi-permeable materials. In the interest of evidence based practice, these options must be independently assessed before they become acceptable as standard-of-care solutions. Generally, very few interventions in the field of limb prosthetics have been investigated by adequately powered randomized control studies, which is a notable detriment to the evidence base of the field. As a consequence, many routinely applied clinical solutions are informed only be anecdotal evidence, manufacturer claims, and subjective assessments with all the associated limitations. Protocols like the one described here are needed to overcome those shortcomings.

### Trial status

Funding for this research is provided by the United States Department of Defense under Award number W81XWH-17-1-0700. The protocol was last updated (version 4) on 8 May 2019. Recruitment began upon receiving ethics approval on 10 December 2018 and is currently ongoing with a planned end date of July 2020.

## Supplementary information

**Additional file 1.** SPIRIT 2013 Checklist: Recommended items to address in a clinical trial protocol and related documents*.

## Data Availability

Not applicable.

## Abbreviations

2MWT: Two Minute Walk Test
HIPAA: Health Insurance Portability and Accountability Act of 1996
ICC: Intraclass correlation coefficient
PCM: Phase-change material
PEQ: Prosthesis Evaluation Questionnaire
SPIRIT: Standard Protocol Items: Recommendations for Interventional Trials
